# Epigenetic perturbations in the pathogenesis of mustard toxicity; hypothesis and preliminary results

**DOI:** 10.2478/v10102-010-0048-5

**Published:** 2010-11

**Authors:** Ahmet Korkmaz, Hakan Yaren, Z. Ilker Kunak, Bulent Uysal, Bulent Kurt, Turgut Topal, Levent Kenar, Ergun Ucar, Sukru Oter

**Affiliations:** 1 Department of Physiology, School of Medicine, Gulhane Military Medical Academy, Ankara, Turkey; 2 Department of Nuclear, Biologic and Chemical Warfare, Gulhane Military Medical Academy, Ankara, Turkey; 3 Department of Pathology, School of Medicine, Gulhane Military Medical Academy, Ankara, Turkey; 4 Department of Pulmonary Medicine, School of Medicine, Gulhane Military Medical Academy, Ankara, Turkey

**Keywords:** mechlorethamine, toxicity, epigenetic, histone acetylation, DNA methylation

## Abstract

Among the most readily available chemical warfare agents, sulfur mustard (SM), also known as mustard gas, has been the most widely used chemical weapon. SM causes debilitating effects that can leave an exposed individual incapacitated for days to months; therefore delayed SM toxicity is of much greater importance than its ability to cause lethality. Although not fully understood, acute toxicity of SM is related to reactive oxygen and nitrogen species, oxidative stress, DNA damage, poly(ADP-ribose) polymerase (PARP) activation and energy depletion within the affected cell. Therefore several antioxidants and PARP inhibitors show beneficial effects against acute SM toxicity. The delayed toxicity of SM however, currently has no clear mechanistic explanation. One third of the 100,000 Iranian casualties are still suffering from the detrimental effects of SM in spite of the extensive treatment. We, therefore, made an attempt whether epigenetic aberrations may contribute to pathogenesis of mustard poisoning. Preliminary evidence reveals that mechlorethamine (a nitrogen mustard derivative) exposure may not only cause oxidative stress, DNA damage, but epigenetic perturbations as well. Epigenetic refers to the study of changes that influence the phenotype without causing alteration of the genotype. It involves changes in the properties of a cell that are inherited but do not involve a change in DNA sequence. It is now known that in addition to mutations, epimutations contribute to a variety of human diseases. Under light of preliminary results, the current hypothesis will focus on epigenetic regulations to clarify mustard toxicity and the use of drugs to correct possible epigenetic defects.

## Introduction

Among the available chemical warfare agents, sulfur mustard (SM), also known as mustard gas, has been a widely used chemical weapon. Because of its devastating toxicity, its use during the World War I earned it the sobriquet “king of the battle gasses”. Other compounds such as nitrogen mustard (HN2) were developed during World War II, but found to be unsuitable as a munition (Smith and Skelton, 2003; Kehe and Szinicz, 2005). Soon after discovering HN2, it became the first non-hormonal agent used in cancer chemotherapy. A number of nitrogen mustard derivatives such as cyclophosphamide (CP), ifosfamide (IF), mechlorethamine, melphalan and chlorambucil are valuable cytotoxic and radiomimetic agents for the treatment of cancer (Kehe and Szinicz, 2005).

## Proposed mechanism of acute toxicity

SM is absorbed by inhalation or through the skin following exposure. Potent alkylating activity is not a result of mustards themselves but is due to their derivatives including sulfonium and carbonium for SM, and aldophosphamide and acrolein for CP. These derivatives are also responsible for the side effects of chemotherapeutic mustards. After absorption, SM undergoes intramolecular cyclization to form a sulfonium or carbonium intermediate. This, in turn, reacts with and alkylates nucleic acids and proteins, resulting in impaired cell homeostasis and eventual cell death. Oxidative and nitrosative stress contribute to the early effects of SM poisoning. It typically affects 3 major organ systems: skin, lungs, and eyes. When absorbed in large amounts it can also damage rapidly proliferating cells of the bone marrow and cause severe suppression of the immune system, as well as other systemic toxicities such as neurologic and digestive disorders.

After several decades of research it was revealed that CP and other toxic agents share most of the same pathophysiologic mechanisms (Korkmaz *et al*., 2006; Korkmaz *et al*., 2007). Recent data consistently proves that reactive oxygen species (ROS) (Yildirim *et al*., 2004; Ozcan *et al*., 2005; Sadir *et al*., 2007) as well as reactive nitrogen species (RNS), for instance excessive amounts of nitric oxide (NO) produced by inducible nitric oxide synthase (iNOS) (Korkmaz *et al*., 2003; Oter *et al*., 2004; Topal *et al*., 2005; Ucar *et al*., 2007), involve in initial detrimental effects of all mustards. Currently, available knowledge supports the idea that a major cause of the toxicity of SM as well as other mustards is the formation of enormous amounts of the highly toxic reactant, peroxynitrite (ONOO^−^), (Korkmaz *et al*., 2005; Yaren *et al*., 2007). Thus, both oxidative and nitrosative (nitro-oxidative) stress take place in pathophysiology of acute mustard toxicity.

A direct toxic effect of ONOO^−^ at the site of its production involves an intriguing process which decides the fate of cells. ONOO^−^ is *per se* not a radical but is a powerful nitrosating agent. ONOO^−^ interacts with and covalently modifies all major types of biomolecules including membrane lipids, thiols, proteins and DNA (Demicheli *et al*., 2007; Pacher *et al*., 2007). ONOO^−^ activates matrix metalloproteinases (MMPs) (Okamoto *et al*., 2001; Wu *et al*., 2001) and triggers the expression of selectins and cellular adhesion molecules, via enhancing of NF-κB activation (Szabo 2003), thereby promoting pro-inflammatory responses.

The mutagenic properties of ONOO^−^-induced modified products have also been determined (Juedes and Wogan, 1996; Whang *et al*., 1998). Several studies have shown that NO itself does not induce DNA single-strand breaks *in vitro* in plasmid DNA (Tamir *et al*., 1996; Masuda *et al*., 2002), whereas exposure of plasmid DNA to pre-formed ONOO^−^ (Yoshie and Ohshima 1997) or NO plus O_2_·^−^ generated concurrently (Chaturvedi *et al*., 1998) induces DNA strand breaks. Single strand breakage can be induced by treatment with very low concentrations of ONOO^−^ indicating that this agent is a potent inducer of this type of damage to DNA (Yermilov *et al*., 1996). These observations suggest additional pathways by which ONOO^−^ may be associated with not only elevated DNA damage but also impairment of DNA repair capacity (Chien *et al*., 2004). ONOO^−^ induces apoptosis and necrosis in cells. More highly elevated exposure of this agent is associated with necrosis rather than with apoptosis (Szabo, 2003; Virag *et al*., 2003). In this mechanism, activation of the DNA repair enzyme poly(ADP-ribose) polymerase-1 (PARP-1), a member of PARP enzyme family, mediates ONOO^−^-induced necrosis. PARP-1 detects and signals DNA strand breaks induced by a variety of genotoxic insults. Upon binding to DNA, strand breaks occur and, PARP transfers ADP-ribose units from the respiratory coenzyme nicotinamide adenine dinucleotide (NAD^+^) to various nuclear proteins. From a physiological view point, PARP-1 activity and poly(ADP-ribosyl)ation reactions are implicated in DNA repair processes, the maintenance of genomic stability, the regulation of gene transcription, and DNA replication. An important function of PARP-1 is to allow DNA repair and cell recovery under conditions associated with a low level of DNA damage. In case of severe DNA injury, overactivation of PARP-1 depletes the cellular stores of NAD^+^, an essential cofactor in the glycolytic pathway, the tricarboxylic acid cycle, and the mitochondrial electron transport chain. As a result, the loss of NAD^+^ leads to a marked reduction in the cellular pools of ATP, resulting in cellular dysfunction and cell death via the necrotic pathway (Szabo, 2003; Virag *et al*., 2003). This is known as “suicide hypothesis” of PARP activation and seems to be a regulatory mechanism to eliminate cells after irreversible DNA injury. Experimental evidence has established that the PARP-1 pathway of cell death plays a pivotal role in tissue injury and organ dysfunction in CP-and SM-induced toxicity (Kehe *et al*., 2008; Korkmaz *et al*., 2008b). Cells that are intoxicated by SM and are repaired by the PARP-1 seem to be responsible of the delayed toxicity. These cells should be free of major DNA damage, are able to divide but they also have either light to mild but not severe DNA damage and/or other type of damages.

Unfortunately, it is not clear how mustard gas causes severe multi-organ damage years after even a single exposure. It is well known that most metabolites of mustard agents are excreted in the urine within a few weeks after exposure (Somani and Babu, 1989). It is also well documented that mustard analogues such as CP and IF severely damage DNA and other molecules, and have toxicity long after the initial exposure leading to cell death and an increased likelihood of cancers (Smith *et al*., 2003). As noted above, the initial toxicity of mustards relates to a massive onslaught of highly reactive oxidizing and nitrosating molecules. For most mustard agents, once these changes occur the cellular effects essentially disappear. For SM, however, there are delayed progressive effects which render victims incapacitated for years (Balali-Mood *et al*., 2005; Hefazi *et al*., 2005; Mahmoudi *et al*., 2005; Balali-Mood and Hefazi, 2006; Shohrati *et al*., 2007). The pathophysiologic mechanism of delayed mustard gas toxicity currently has no clear mechanistic explanation.

## Epigenetic perturbations; possible explanation of SM-induced delayed toxicity

If the nuclear DNA in a cell is damaged, it is either repaired via several means including DNA repair enzymes (Nohmi *et al*., 2005) or the cell eventually dies (Szabo *et al*., 2001; Szabo, 2003). However, if SM causes not only genotoxicity but also it alters epigenetic processes, this could explain, at least in part, the delayed effects of this warfare agent. We propose that, the epigenetic regulation of the DNA may be the underlying mechanism of delayed effects of SM (Bird, 2002; Lyko and Brown, 2005).

A genetic change is thought of as a permanent, inheritable change affecting every cell if it is passed along through the germline. However, these assumptions are not totally correct. In addition to the DNA inheritance system underlying classical genetics, it is now recognized that variations can be transmitted between generations in other ways; the epigenetic inheritance system (EIS). The traditional view that gene and environment interactions control disease susceptibility can now be expanded to include epigenetic reprogramming as a key determinant of origins of human disease (Bronner *et al*., 2007). The term epigenetic describes the study of heritable alterations in gene expression that occur in the absence of changes in genome sequence. This can be contrasted with genetics, which deals with the transmission of information based on differences in DNA sequence. Therefore, epigenetic gene regulation requires molecular mechanisms that encode information in addition to the DNA base sequence and can be propagated through mitosis and meiosis. Our current understanding of epigenetic gene regulation involves two classes of molecular mechanisms: DNA methylation and histone modifications (Bestor, 2000; Yang and Seto, 2007).

The chromatin structure is influenced by DNA methylation and DNA-histone interactions. The DNA-histone interaction is further influenced by covalent modification of histones and the action of DNA-binding proteins. The epigenotype can be transmitted from parent cell to daughter cell maintaining a specific epigenotype within cell lineages. Thus, the phenotype is a result of the genotype, the specific DNA sequence, and the epigenotype. The genotype must exist in a particular chromatin configuration, the epigenotype, which allows a secondary level of fine control over gene expression. EIS is generally accepted less stable than the genetic system, and more sensitive to environmental (McLachlan 2001), nutritional (Gallou-Kabani and Junien, 2005) and chemical toxicants (McLachlan *et al*., 2001; Bombail *et al*., 2004). Epigenetic memory of cells can be passed on to subsequent generations and can transfer the perturbed epigenome upon unaffected or normal genetic sequences. The epigenotype shows far greater plasticity than the genotype, and it has been speculated that epigenetic errors could be a major contributor to human diseases (Jiang *et al*., 2004).

**Figure 1:**
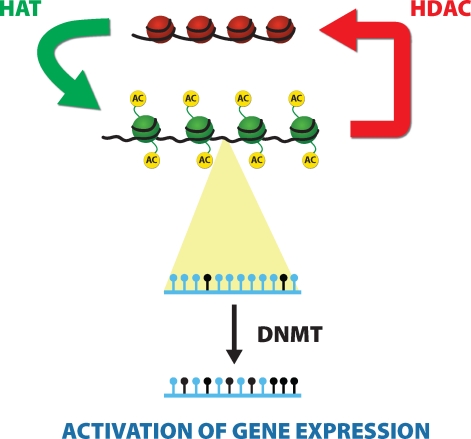
Our current understanding of epigenetic gene regulation involves basically two classes of molecular mechanisms: DNA methylation and histone modifications. A variety of enzymes are involved in this process including most importantly DNA methyltransferases (DNMTs), histone deacetylases (HDACs) and histone acetyl transferases (HATs). Indeed, the transcriptional status of all genes (silent, repressed or active) is determined by its chromatin environment and many molecular responses to toxicants involve alterations in gene expression that are elicited via changes in the chromatin structure of target genes. The steady state level of histone acetylation is regulated by the HATs and HDACs. HATs are responsible for the addition of acetyl groups that stabilize open chromatin structures, while the HDACs deacetylate histones, and are thus responsible for resetting chromatin into a close conformation. DNA methylation is mediated by several proteins. DNMTs add methyl groups to the cytosines in CpG dinucleotides. Three active DNMTs have been recognized in humans and are designated DNMT1, DNMT3a, and DNMT3b. Each DNMT may have a specific role in the methylation process, or may act in association with another methyltransferase. Open chromatin is considered as transcriptionally active, whereas condensed chromatin is transcriptionally inactive.

A variety of enzymes are involved in this process including most importantly DNA methyltransferases (DNMTs), histone acetyl transferases (HATs) and histone deacetylases (HDACs) (Miremadi *et al*., 2007). Indeed, the transcriptional status of all genes (silent, repressed or active) is determined by its chromatin environment and many molecular responses to toxicants involve alterations in gene expression that are elicited via changes in the chromatin structure of target genes (Figure 1).

Since the genome contains information in two forms, genetic and epigenetic, become clear, initial studies focused on human cancers and rapidly revealed that most of human cancers are relevant to epigenetic aberrations (i.e., epimutations) (Ducasse and Brown, 2006) including epigenetic silencing (Robertson, 2001) of tumor suppressor genes because of hypermethylation (Esteller, 2007). To date, numerous tumor suppressor genes have been found to undergo hypermethylation in cancer (Garinis *et al*., 2002; Das and Singal, 2004). Such epimutations rarely appear in healthy tissue, indicating that epigenetic therapies may have high tumor specificity. Currently, two DNMT inhibitors (DNA demethylating drugs) received US Food and Drug Administration approval for the treatment of myelodysplastic syndrome: 5-azacytidine (Vidaza®) and its derivative decitabine (Dacogen®) are now being marketed (Brueckner *et al*., 2007) and several presently available drugs are under extensive clinical investigations (Peedicayil, 2006).

Exposure to mustards may trigger a variety of mechanisms along with nitro-oxidative stress, inflammation and DNA damage. If this is the case, a number of drugs (e.g., anti-oxidants, anti-inflammatory agents) in treatment of experimental toxicity may not be beneficial for victims. Data based on the experience in Iranian veterans exposed to the agent during the Iran-Iraq conflict (1983–88) have clearly shown that toxicity of SM is almost incurable even extensive treatments (Balali-Mood and Hefazi, 2005). It was described that the toxic effects of SM poisoning in a group of 40 severely intoxicated Iranian veterans, 16–20 years after their initial exposure. The most commonly affected organs, in this study, were lungs (95%), skin (75%) and eyes (65%) (Balali-Mood *et al*., 2005). Another clinical study revealed that the delayed toxicity of SM persists on the respiratory tract (78%), central nervous system (45%), skin (41%) and eyes (36%) in 236 Iranian veterans in between 2 and 28 months after exposure (Balali-Mood and Hefazi, 2006). In a study by Khateri *et al*., (2003) on 34,000 Iranians, 13–20 years after exposure to SM, the most common complications were still found in the lungs (42.5%), eyes (39%), and skin (24.5%) (Khateri *et al*., 2003). Although, a vast array of experimental remedies, there is no consensus on medical management of victims exposed to mustard gas, other than thorough decontamination and supportive care. Therefore, this paucity of information regarding the medical management warrants novel approaches to the pathogenesis of SM poisoning. We recently reviewed the possible epigenitc mechanisms involved in the pathogenesis of mustard toxicity (Korkmaz *et al*., 2008a).

## Preliminary study of the hypothesis

**Figure 2:**
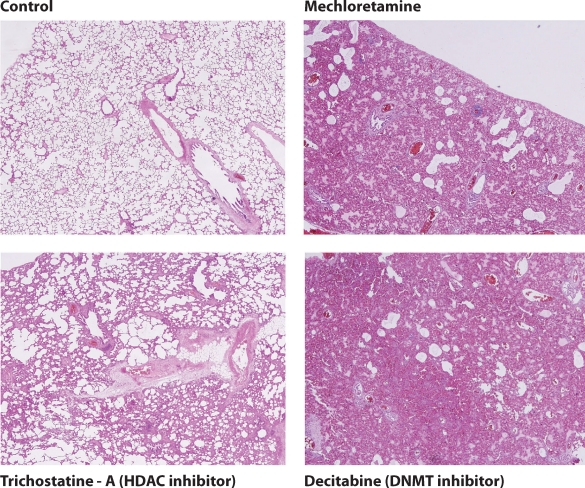
Representative histological pictures of the preliminary study groups. Normal lung tissue of rat (control). Alveolar cavities are normal; there is no edema and hemorrhage (control). In MEC group, interstitial edema and some hemorrhage are seen in several areas. Inflammatory cell infiltration is present in the alveolar cavity, mucosal epithelium and airways. Administration of HDAC inhibitor alleviated the MEC symptoms. The alveolar cavities, vascular bed and airways are relatively normal. In the representative figure of the DNMT inhibitor group interstitial edema and hemorrhage as well as septal thickening is apparent in many areas. Many airways are larger than those of the control group and similar to MEC group (Hematoxylin & Eosin × 40).

The experimental protocol was approved by the animal ethical committee of Gulhane Military Medical Academy. A total of 40 male SD rats were divided into 4 groups. Group 1 served as control and given 2 ml saline, three groups received single dose of mechlorethamine (MEC) (3.5 mg/kg subcutaneously) with the same time intervals. Group 2 received MEC only; group 3 received histone deacetylase (HDAC) inhibitor (Trichostatine A, Sigma Germany) (1 mg/kg) and group 4 received DNA methyl transferase (DNMT) inhibitor (decitabine, Sigma Germany) (0.02 mg/kg), intraperitoneally. MEC injection resulted in severe lung toxicity with strong interstitial and alveolar edema, hemorrhage, emphysematous changes as well as mild inflammatory cell infiltration and septal thickening. In group 3, the HDAC inhibitor significantly reduced interstitial and alveolar edema, hemorrhage and inflammatory cell infiltration. On the other hand, we have observed severe lung damage by using DNMT inhibitor (group 4). In HDAC inhibitor group, the results were close to sham group. In DNMT inhibitor group, however, histology of lungs was worse than MEC group results (Figure 2).

These preliminary results revealed that, MEC itself and/or its intracellular metabolites perturb the epigenetic environment of the affected cell in lung tissue. Hypothetically, MEC may cause HDAC induction leading to a variety of gene silencing. Since the animals were healthy and free of disease, inhibiting HDAC by Trichostatine A means that, mustards may activate HDAC which results in silencing a variety of beneficial genes which code, for example, anti-oxidant enzymes and anti-inflammatory proteins. Since decitabine worsen the MEC-induced lung injury, inhibition of DNMT may silence the genes those are physiologically silenced but require methylation to be activated. Although these genes have normally lower activation level or totally inactive, any proper stimulus can cause gene activation. From this manner, it seems logical that, by inhibiting DNMT, we may block gene expression such as anti-oxidant enzymes and anti-inflammatory proteins. If this mechanism is true, inhibiting HDAC may block the silencing of beneficial genes, on the contrary inhibiting of DNMT may result the silencing of the same group genes. Further studies are needed to clarify the involvement of epigenetic perturbations in the pathogenesis of mustard toxicity.

## Concluding remarks

It seems that epigenetic modifiers have influence on gene expression in the pathogenesis of mustard-induced lung toxicity. Epigenetic therapy is a new and rapidly developing area in pharmacology. To date, most trials of epigenetic drugs have been conducted to evaluate their effects on cancers, many of which have shown promising results. Epigenetic drugs alone or in combination with conventional drugs may prove to be a significant advance over the conventional drugs used to treat both acute and delayed SM toxicity. Since epigenetic defects are thought to underlie a broad range of diseases, the scope of epigenetic therapy is likely to expand. At present, the targets for epigenetic drugs are DNMTs and HDACs, but since a variety of molecules are involved in epigenetic mechanisms, there are several other targets as well.

Collectively, we may speculate that, mustards may cause epigenetic perturbations within the affected cells and reducing mustard-induced lung toxicity requires gene activation rather than gene silencing. This preliminary observation warrants future studies to clarify the epigenetic perturbations in the pathogenesis of delayed-mustard toxicity. A variety of HDAC inhibitors as well as other epigenetic modifiers could give valuable results in experimental studies and may open new avenues for treatment of SM-induced toxicity.
